# Quality Standards and Contractual Terms Affecting Food Losses: The Perspective of Producer Organisations in Germany and Italy

**DOI:** 10.3390/foods12101984

**Published:** 2023-05-13

**Authors:** Roberta Pietrangeli, Ronja Herzberg, Clara Cicatiello, Felicitas Schneider

**Affiliations:** 1 Department of Economics, Engineering, Society and Business Organization (DEIM), University of Tuscia, Via del Paradiso 47, 01100 Viterbo, Italy; 2 Thünen Institute of Market Analysis, Bundesallee 63, 38116 Braunschweig, Germany; ronja.herzberg@thuenen.de (R.H.); felicitas.schneider@thuenen.de (F.S.); 3 Department for Innovation in Biological, Agro-Food and Forest Systems, University of Tuscia, Via san Camillo de Lellis snc, 01100 Viterbo, Italy; cicatiello@unitus.it

**Keywords:** food loss and waste, fruit and vegetables, supply chain structure, content analysis, postharvest, qualitative interviews, supplier/retailer interface, bargaining power

## Abstract

With the aim of disclosing the antecedents and dynamics of food loss generation in the upstream stages of the fruit and vegetable sector, this paper presents the results of a series of semi-structured interviews with 10 Producers’ Organisations (POs) in Germany and Italy. The content of the interviews is analysed by applying a qualitative content analysis approach, thus disclosing the most relevant issues affecting food loss generation at the interface between POs and buyers (industry and retailers). Several similarities emerge as we compare the answers provided by Italian and German POs, especially concerning the role of retailers’ cosmetic specification on products in the generation of losses. Instead, the structure of contracts regulating commercial transactions between POs, industry, and retailers show noticeable differences, apparently resulting in a greater capacity to plan the demand of products from the beginning of the season in the Italian context. Despite these differences, this study confirms the key role of POs in increasing farmers’ bargaining power against the buyers, both in Germany and Italy. Further research is needed to compare circumstances in other European countries and to analyse why the similarities and differences identified occur.

## 1. Introduction

Food loss (FL) occurs due to several factors and inefficiencies in the supply chain during the early stages of the food supply chain, from harvest up to, but not including, the retail level [1,2,3]. It is estimated that in the harvest and postharvest process, FL sums up to 14% [2] of production, while 17% of food available for consumption is wasted in the downstream stages of the supply chain [4]. FL can refer to a reduction in the mass of food destined for human consumption (quantitative losses), or to qualitative losses, referring to a decrease in food attributes that reduces the nutritional value of products or its economic value due to noncompliance with quality standards [2]. The Sustainable Development Goal 12.3 [5] states that FL should be reduced by 2030, and the progress towards this objective is measured with the Food Loss Index [6]. However, in the early stages of the value chain data is still scarce [7], and the dynamics of FL generation in the interface between suppliers (producers and their organisations) and buyers (retailers and industry) are still being studied.

Focusing on FL in the fresh fruit and vegetable (FFV) supply chains, FAO reports that between 20 and 25% of produce is lost from postharvest to distribution worldwide [2]. In this sector, producers’ organisations (POs) play a crucial role. These are associations of farmers, which take charge of the postharvest phase by collecting, storing, processing, and selling the products, thus strengthening the farmers’ collaborative bargaining power [8]. In the European Union, POs are legally recognized and financially supported [9]. Their aim is to bridge farmers with buyers by concentrating the supply of agricultural commodities, negotiating the contracts, jointly planning the production, and supporting farmers with technical advice and help in machinery investments [8,10,11,12]. By aggregating the production of farmers, POs try to contrast the higher power of buyers during the negotiation of contracts, taking charge of the economic risk related to these transactions [13,14,15].

Prices, quality and production standards, logistics, delivery, payment schedules, promotions, risk sharing, and many other details are typically agreed upon in supply contracts established between POs and buyers [16]. These contracts usually include conditions on the quantities and delivery times imposed by buyers, as well as cosmetic standards that the products must fulfil [17]. These standards for commercialization may either be imposed by regulations to assure food safety, or applied by buyers to standardize the appearance and features of the products [18,19]. The latter, which are referred to as ‘private marketing standards’, are particularly relevant in the FFV sector. Retailers set strict quality standards under the hypothesis that customers would reject or have a lower willingness to pay for suboptimal products [18,20]. Such standards create a bottleneck for products entering the market, thus generating significant quantities of suboptimal products which have the same intrinsic quality and safety of ’accepted’ products, but are not conforming with cosmetic standards (for example mass, shape, size, etc.), or are approaching the use-by date labelled on the packaging [18]. In the absence of alternative channels for commercialization, these products are likely to become FL [21]. Moreover, due to the perishable character of (most) FFV, once these products are rejected for cosmetic reasons, the redirecting to alternative channels is hardly feasible because they would spoil in the meanwhile. While there is a growing body of literature on how suboptimal products might be managed in the retail sector [19,21,22], there is a lack of knowledge about how the implementation of private marketing standards affects the other key players of the upstream supply chain, i.e., POs and, in turn, the affiliated producers.

Porter et al. [23] report that the range of losses of FFV due to cosmetic standards varies between 4 and 58% in the European Economic Area, excluding preharvest grade-out losses. This data is supported by other studies that estimate a loss of up to 40% of harvested FFV due to cosmetic standards requested by the retailers [24,25,26]. One of the few studies reporting a direct measurement monitored persimmon losses in Spain, indicating that 16.11% of delivered persimmon quantity is downgraded at the cooperative warehouse due to quality standards, and is not paid to primary producers [27]. Willersinn et al. [28] estimated that at the agricultural stage, losses of fresh potatoes were reduced by 3–5% on a total of 53–55% in all the supply chain when lower cosmetic standards were applied; this reduction was even more significant in the processing stage, where a lower standard could lead to a reduction of FL up to 11%.

However, data on FL and the dynamics of its generation in the upstream stages of the supply chain is still scarce [7]. This paper aims to contribute filling this gap by studying how FL is generated at the interface between farmers, POs, and buyers, that is, the harvest and postharvest stages of the FFV supply chain. This paper focuses on the perspective of POs, which represent a main actor in this phase of the supply chain. We compare the processes in relation to private quality standards and contractual terms that lead to FL in the value chain, at the PO level, and across Italy and Germany. Although there is literature available for some countries, such as Germany, the unique contribution of this paper is to provide insights on a new country, Italy, where little information is available, comparing Italy to a better-assessed country. To do so, semi-structured interviews have been conducted with seven German POs and three Italian POs. A standard guideline has been applied, using the MAXQDA program, to conduct the content analysis of the interviews, to make the method comprehensible, and to allow comparison of the two studied countries. The results are analysed in the framework of a structured qualitative content analysis to assess similarities and differences in food loss occurrence between Germany and Italy in the FFV supply chain.

The specific objective of this paper is to address the following research questions: (i) what are the similarities and differences in the market dynamics between POs and buyers in the FFV supply chain in Germany and Italy?; (ii) what are the reasons why FL is generated at the interface between POs and buyers in Germany and Italy?; (iii) do private marketing standards, set by buyers, influence FL in Germany and Italy, and how are the products managed if they do not meet these requirements?

## 2. Materials and Methods

### 2.1. Data Acquisition

A qualitative research approach was chosen to investigate FL and how it is connected to private marketing standards from the perspective of the POs. 52% of POs in Europe operate in the FFV sector. France, Germany, and Italy are the countries where POs are more present: Germany has 692 POs, among which, 31 are in the FFV sector, while Italy has 583 and 315 FFV POs, respectively [29].

We chose the method of semi-structured interviews to shed light on the mechanisms and processes related to FL at the PO level. In Germany and Italy, the interviewees were selected based on a list of POs working in the FFV sector within the country, making sure to cover different crop types and regions. A similar methodological approach was applied in both countries, with the aim to jointly analyse and compare the results. The interviewees were persons responsible for sales and marketing, for quality control, or for the management of the POs.

A guideline was developed for the interviews in Germany and Italy, addressing the same issues to contribute to the objectivity and trustworthiness of the study [30]; all interviews covered the following topics:

1. Causes of FL in the supplier/retailer interface;
2. Design of contracts and arrangements that POs have in place with farmers and retailers;
3. Relation between the structure of contracts, orders’ management, and quantity of FL;
4. Quality management process along the supply chain and criteria that influence the decisions;
5. Management of products that do not meet the required quality standards.

Interviews in Germany were conducted between October 2020 and January 2021, and were partially analysed by Herzberg et al. [31], targeting the issue of food loss creation through unequal bargaining power, trading practices, product specifications, orders, and contracts in Germany. Interviews in Italy were conducted between September and December 2022.

The interviews were conducted face-to-face, whenever COVID-19 restrictions allowed for, or online/via telephone. In total, seven interviews were conducted with German POs and three with Italian POs. Although the number of interviews with POs conducted in Italy is lower, the selected POs are very relevant in the national market; all together, they produce more than 5% of the national production of fennel and potatoes; all in all, they reach a turnover of almost EUR 300 million. Table 1 provides the description of the POs involved in the study.

Interviews in Germany were recorded and literally transcribed. Interviews held in Italy were not recorded, but full comprehensive notes were taken on the same day of the interview.

### 2.2. Data Analysis

We applied a structured content analysis approach for qualitative data, inspired by Kuckartz et al. [32], to the interviews. The MAXQDA software was fed with transcriptions for Germany and notes for Italy from the interviews.

In the applied methodology, a coding system was created and used to sort all the statements of the interviews. It is based on a deductive and inductive coding. The deductive codes are rooted in the theories and literature described in the introduction section of the paper. The inductive codes were developed during the analysis of the material of the German interviews. The same coding system was then applied to the notes of the Italian interviews.

## 3. Results and Discussion

**Figure 1:**
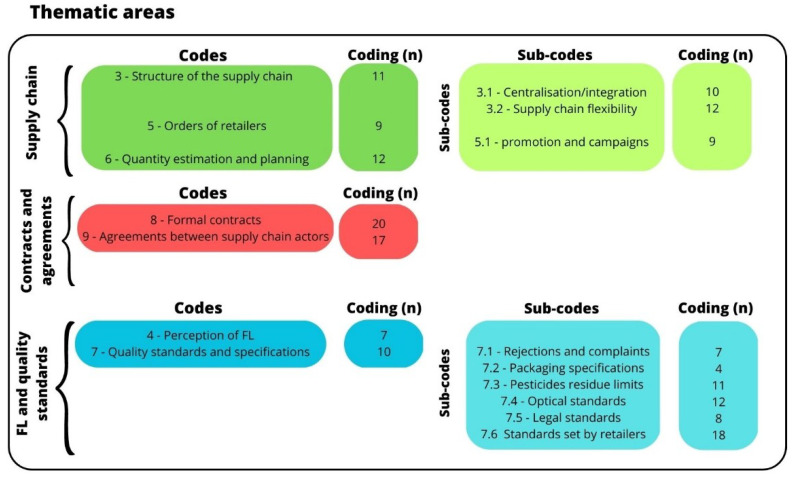
Structure of the coding systems developed and applied in the content analysis to sort the single statements (i.e., codings; *n* = 177). The codings are divided into three thematic areas: supply chain (*n* = 63), contracts and agreements (*n* = 37), and FL and quality standards (*n* = 77). Every thematic area has different codes, meaning different categories to sort the codings; some categories are also divided into subcategories (subcodes).

As a result of the content analysis on the interviews, two different tables were developed: a quantitative cross table, and a qualitative segment matrix coding the data of the interviews. The results of the quantitative cross table are summarized in Figure 1. In total, we have 177 codings, divided into 3 thematic areas: the answers dealing with supply chain organisation (with 63 codings), followed by the issues related to contracts and agreements (with 37 codings), and lastly the FL and quality standards (with 77 codings). These three areas are reflected in the next subsections of the paper, where the results are reported and discussed separately for each topic.

In the qualitative segment matrix, codes are listed in the rows, and the statements of each PO are reported in the columns. The segment matrix allows for the comparison of similarities and differences between the answers of the different POs on the same category in a qualitative manner. Therefore, it helps to compare POs and countries based on the selected codes and subcodes. Table 2 reports the main similarities and differences between German and Italian POs emerging from the interviews, which are organised along the three thematic areas represented in Figure 1.

### 3.1. Supply Chain Organization

The content of the interviews confirms the central role of POs in the value chain, both in Germany and in Italy. Interviewees describe the POs as intermediary between farmers and retailers, collecting and storing the FFV products from farmers, negotiating contracts with retailers, and taking charge of logistics and deliveries upon retailers’ orders. When products are harvested, farmers bring them to the PO, where they are stored or directly delivered to retailers.

The length and organisation of the process at the surveyed POs depend on the type of fruit and vegetables; super fresh products such as strawberries, salads, and other leafy vegetables are quickly delivered to retailers, which is in line with the findings from Surucu-Balci et al. [33]. In contrast, other products are typically stored in the warehouse of the PO until the order of the buyer. Some differences are detected in the structure of the postharvest operations across the two countries. In Italy, POs take charge of selection and packaging of products before delivering it to the buyers; these operations take place in the warehouse of the PO. Instead, in Germany, many items (for example cabbages and salads) are selected and packed directly in the field. Therefore, a selection and sorting out of unsuitable produce already happens in the field, while in Italy the selection mostly happens at the PO premises. This is particularly interesting in relation to FL generation, because the different organisational patterns influence where FL is generated. It therefore also defines the actor of the supply chain in charge of managing such losses [34], as well as the available options for recovery of the discarded items [35].

During the interviews, the structure of the FFV supply chain emerges as being vertically integrated. In general, POs plan their production together with farmers and they manage the quantity to be delivered by different farms, considering the changing environmental conditions. As also confirmed by the literature [36], these efforts are intended to reduce the volatility of prices in the FFV market. According to our study, Italian POs plan at the beginning of the season where to cultivate each item, based on the quantity they expect to deliver to the retailers during the upcoming season, and assuring a proper crop rotation in the fields. As argued by Aschemann-Witzel et al. [37], this approach assures a better management of the surplus products, giving the PO the possibility to reallocate surplus or suboptimal products to different marketing channels. In Germany, the process is basically the same, although the German sample comprises, on the one hand of POs that work rather top-down and, on the other hand, of others that work less vertically integrated, where members have more leeway in product and quantity planning.

The interviewees disclosed that in both countries POs also store harvested products, especially those with a long shelf life. These items remain in stock until the PO receives an order from a retailer; orders are typically made with short notice (AxA, today for today; or AxB, today for tomorrow). The flexibility that is required in this situation, besides being a logistical challenge, can also affect FL levels, as also confirmed by Hobbs et al. [38].

The interviews also suggested that a main challenge for the POs is the estimation of the quantity of products they will need during the season to satisfy the orders. In Italy, the quantities to be delivered are agreed upon among POs and buyers at the beginning of the season. In Germany, quantities are informally agreed upon within annual consultations. Last minute changes in procurement decisions by retailers can always occur, considering peaks or off-peaks of consumer demand for that product. This could lead to sudden surplus stocks and bring prices down, causing FL as a consequence in our results, and suggested by Bustos et al. [39].

### 3.2. Contracts and Agreements

The role of POs as a bridge between farmers and the market is very clearly expressed in the interviews. POs represent the farmers in the commercial relations with the buyers of different marketing channels (retailers, food industry, and wholesale trading centres). The interviewees confirmed that buyers may take advantage of their bargaining position to minimize the economic risks emerging from the transaction [31,40]. To cope with this situation, POs usually have contracts with different buyers, and among them, with different retailers. This evidence is in line with Fałkowski et al. [41], who report that POs are recognized to improve farmers’ position against the industry and retail sector. In addition, the larger the POs´ size, the greater the benefits offered to their members, also in terms of profits, lower costs, and services [42]. German interviewees report that POs sometimes pass the economic risk onto the upstream farmers, e.g., by passing potential monetary discounts due to low product quality onto them. This does not happen in POs in the Italian sample.

The relations between the farmers and the POs are usually regulated through written contracts in the Italian POs surveyed. In this case, the product is owned by the PO from the beginning of the season. Instead, in Germany, there are different patterns: either the PO may buy the product from the farmer, as it happens in Italy, or the PO only acts as an intermediary between the farmer and the retailer, and the ownership of the product remains with the farmer until the retailer buys the product. What makes these German contracts different is the ownership of the goods, which is managed differently, and this also influences the responsibility of dealing with returned/rejected produce.

In most cases, POs negotiate contracts both with the industry and retailers, as well as with further potential buyers. The contracts with the food industry are generally considered more reliable by the Italian interviewees because, as one of them said, “*what they write in the contracts is what they ask for, products hardly remain in the field*”. In Italy and Germany, food industries hardly change the quantity demanded during the year, thus allowing for a better planning of crops. However, the price paid by retailers is higher than the price paid by the food industry. Therefore, POs must carefully find a balance between these kinds of contracts, considered as safer, and the more remunerative ones, signed with retailers. For this reason, as also suggested by Camanzi et al. [43], larger POs tend to have larger volumes of products contracted with retailers than with food industries, in order to have higher remuneration. Instead, POs with fewer members, and thus smaller in size, tend to have more contracts with food industries, to guarantee the sale of products. However, selling fresh FFV to the retailing sector remains the first choice due to the substantially higher economic margins. It should also be considered that the average acreage per farm and the number of farmers per PO is heterogenous, as shown in Table 1. This makes it difficult to generalize these concepts, as POs composed by smaller farms may produce a broader variety of products that would be less likely to meet the private quality standards required by retailers.

The interviews reveal significant differences in the timing and structure of contracts between POs and buyers across Italy and Germany. In Italy, every contract is discussed and signed at the beginning of the year, which includes the specification of the quantities agreed upon by all the actors within the annual consultation. It also comprises the private quality product standards to be respected, delivery timing, and the schedule of promotions. Instead, in Germany, contracts cover several years, but they only state very general terms of cooperation among the parties, e.g., codes of conduct, working conditions of employees, etc. This means that no binding agreements are made on quantities, prices, crop types, etc. Yearly consultations to negotiate quantities and price corridors do take place in Germany at the beginning of each year, but no formal kind of contract emerges from these consultations, rather, agreements are written on a “*piece of paper*”, as mentioned by one of the German interviewees. Final quantities and prices are defined on a short-term basis. This situation may lead to last-minute changes in the orders of products in both countries. In Italy, the timing of orders and the quantity ordered every day can vary significantly during the season, especially for super fresh products that are harvested and delivered on the same day, although the annual quantity agreed upon within the contract stays the same. In Germany, short-term variations can concern both quantity and prices. German interviewees also reported that the lack of specifications within contracts between POs and retailers may bring the risk of having a surplus of products in the peak period of the season at the POs´ level. This is coherent with other evidence in the literature, suggesting that the behaviour of retailers can influence the amount of losses, not only at their stores, but also at the upstream stages of the supply chain [44,45].

Some of these contracts are on the edge of unfair trading practices, as defined by the recent EU legislation [46] which lists the practices which are banned, unless the parties agree otherwise. For example, the buyer is not allowed to unilaterally change the terms of agreements concerning the frequency, method, place, time or volume of supply or delivery of the products [46]. The directive also states that the short notice cancellation of orders for perishable products allows to assume that the supplier will not be able to find an alternative to market or use such products. Take-back agreements, that is the practice to return unsold products to suppliers, are also considered as unfair, though none of the POs in our sample report to have it in force.

German interviewees also mentioned that sometimes retailers may decide to buy items from another supplier (even from abroad) in the middle of the season. This approach can generate large amounts of losses for domestic suppliers.

The Italian interviews disclosed that contracts also define what happens if delivery time or/and quantity are not respected. In this case, retailers usually apply an additional cost to POs, representing a big fee for every delayed load. However, the same issue was not reported in the German interviews, nor was the problem confirmed in the available literature.

Another issue for contract specification is the price. If the price is not remunerative enough, farmers do not have any advantage to work with POs, and they would rather use the spot-market as individual businesses. The prices in agriculture are very volatile, and this is an issue for farmers [47]. The PO works as an intermediary, aiming to secure the farmer’s interest, but the interviews revealed several differences in how this role is interpreted. In Italy, the price that the PO pays to farmers is set in advance at the beginning of the season. In Germany, a price corridor is agreed upon during the annual meetings, and the contracts only include payment times, terms and conditions: “*That means in week A (..) the prices for week B are set, if necessary, the quantities are also discussed and then in week B there is actually only the dropping of this quantity. (..) That means there are no permanent contracts, the form is more of a planning and agreement*”, as well-described in a German interview. Instead, the price between buyer and PO is contracted during the season. Both in Italy and in Germany, prices between POs and retailers are agreed upon weekly or biweekly, and the price-setting for fresh products is influenced not only by the European market, but also by the world market prices. The efficacy of this mechanism is crucial for the effective operation of the market because the connections between producers, processors, and POs assure market and price stability [48]. One of the main advantages that POs can provide to farmers is the stability of product prices, and therefore of their income in the long term [8], even if in some seasons the farmers may get a lower price through the PO than they would get from other market channels.

The European Union, in the Common Agricultural Policy, includes a range of measures to help mitigate price volatility in agricultural markets, including market interventions, risk management programs, and support for sustainable farming practices. One example is the use of public stocks and intervention purchases to stabilize prices during times of market disruption. On the one hand, these measures can help to prevent sharp price fluctuations that could negatively impact farmers and consumers. On the other hand, the withdrawal of products from the market can create a potential amount of FL [49].

Promotion campaigns are also mentioned by interviewees as a tool that is potentially strategic to avoid FL. In Italy, the promotions are already scheduled by retailers at the beginning of the year: POs know in advance in which week they will be active. In Germany, there is also a planning of promotional campaigns at the beginning of the season. However, retailers announce the estimated quantities of the promotion to POs just a few weeks before they start. On the one hand, an early scheduling of promotions enables POs and farmers to plan the production in advance, thus preventing the generation of FL. On the other hand, spontaneous campaigns can be used as leverage to react to unexpected excess quantities. Promotions imply, within a few days, an increasing demand for products that are sold and bought for a lower price; the estimation of the quantities that are actually requested remains difficult [31]. The Italian interviewees report that when retailers schedule promotions, they mainly consider the expected trend of consumer demand, as well as their own needs in terms of planning marketing actions; instead, they do not consider the seasonal variation of the timing of harvesting. The consequence is that the increasing demand for products by retailers, linked to the start of a promotion, may not match the actual peak of products ready for harvest in the field. With respect to this issue, Hobbs et al. [38] argue that POs should plan crop production and orders very carefully to be able to supply the amount required by retailers, but they should also be able to stay flexible.

Despite the planning efforts, excess production can always occur. The approach to handle these surplus products, which cannot easily be marketed, differs between the surveyed countries. From the Italian interviews, it emerges that during the peak of the season, some POs organize trucks to donate surplus products, which would otherwise be lost, to charities. Indeed, the donation of surplus products can help prevent food loss by redirecting excess, edible food to those who need it, and increase the efficiency of the food system [50]. This is facilitated by the existence of a specific legislative framework which regulates the donation of surplus products to charities in Italy [51]. Instead, planned regular donations from POs on a large scale do not seem to exist in Germany, maybe because charities would tend to be overwhelmed by such large amounts of produce, and the management effort would exceed the capacities of the POs. However, it can be expected that this destination for surplus produce will become more frequent in the future, following the launch of the “European Food Donation Act” to encourage food donations across the continent [52].

### 3.3. FL and Quality Standards

Private quality standards imposed by retailers emerge as a crucial issue related to FL generation in all interviews. Each retailer asks for different specific quality standards that are generally stricter than the quasi-public and public standards imposed by the EU and UNECE. Vandemoortele et al. [53] suggest that retailers systematically apply stricter standards because, in this way, they can obtain products of higher quality by inflicting to producers most of the costs related to the achievement of such quality. This mechanism also generates significant FL in the upstream supply chain [13,21,54].

In Italy, quality standards are mostly specified in the contracts. In Germany, POs explain that retailers rather impose these standards in an informal manner or in a written form, but not within the contracts. POs, however, have their own product profile for each variety that elaborates on characteristics that need to be met. Regardless, they must be complied with, otherwise the products are not accepted during the retailer’s quality control upon delivery, and are sent back to the PO due to insufficient quality. One of the most frequently repeated argument by interviewees is that “*if it was for us* (PO)*, we would have sold everything, but retailers do not want it*”. While there is evidence in the literature that retailers have an impact on consumer decision-making and purchasing behaviour [45], they prefer rejecting suboptimal products rather than taking the risk of remaining with unsold products.

When products are sent back from retailers to POs because they do not meet the quality standards requested, they cause FL in mass and economic terms [14]: POs need to repack and rework products that were rejected by retailers and find different marketing channels that are not as remunerative. As rework is also barely lucrative, POs try to avoid items being sent back from the outset. Depending on the general perishability of the rejected produce and the actual condition (e.g., ripeness), the aforementioned procedure will exceed the available time until the end of edibility is reached and, thus, produce has to be disposed of.

While discussing about different types of private quality standards, the interviewees reported that visual standards are one of the most difficult to be reached. Italian POs underline the importance of the visual aspect for Italian retailers: interviewees said that some retailers want the “*most perfect products, beautiful and clean*”. In addition, many German POs mentioned calibre as one of the main standards contributing to FL generation. The interviewees report that the main argument of retailers to justify these requirements is that the consumers would not buy substandard products. However, the evidence shows that there are existing retailer brands selling suboptimal products on a regular basis, at least in some countries (including Germany, Italy and Austria). Recent research shows that consumer acceptance depends on specific characteristics related to certain products. This means they accept a specific flaw (e.g., a dent) for one product, but not for another [18,55,56,57]. Moreover, there is no evidence that consumers are actually able to recognize small cosmetic differences among products, meaning that in some cases they might not even be able to identify non-compliance with cosmetic standards [58]. Interviewees argue that this would allow for a less strict implementation of cosmetic standards, for example, regarding the size of fruit and vegetables. Meyer et al. [59] suggest that a change from marketing more items per piece to a marketing per mass in Germany would reduce the pressure to produce same-size products. However, in Italy, most FFV products are already sold by mass, and still this issue was raised in the interviews.

Although some studies have detected a negative image effect on retailers due to selling of suboptimal products [60], POs argue that suboptimal products have the “*same organoleptic and quality characteristics and same taste, but they are just ugly*”. This claim is in line with other studies which underline the need for raising awareness among customers [18,58,61], especially adopting some marketing strategies [62].

Since suboptimal products are a big issue at the interface between POs and retailers, alternative marketing channels are available to reallocate products. There are marketing options (e.g., “patata imperfetta” in Italy or “die krummen Dinger” in Germany) to sell such produce at the fresh market by retail chains, but as mentioned above, marketing is challenging due to consumer preferences. Alternative fresh marketing channels could directly address open-minded consumers or public institutions, e.g., by offering suboptimal food boxes using online subscription systems in Germany (German examples are the start-ups Querfeld [63] and Etepetete [64]), Austria (Austrian example is the start-up Afreshed [65]), or the UK (UK example is the start-up Oddbox [66]) or to address food service businesses.

From the perspective of the POs interviewed, limits to pesticide residues also represent a critical issue, because German and Italian retailers ask for stricter thresholds than the ones imposed by EU regulations. Although in Italy they do not seem to affect FL to a great extent, German interviewees are of the opinion that the standards are quite hard to reach and are considered among the most important reasons affecting FL levels. Reasons are the inability to control pests and diseases by applying only small amounts or a restricted number of agents, but also the sorting out of produce exhibiting too high levels and/or too large numbers of pesticide residues.

The challenge seems to be even harder for organic FFV products, where higher restrictions are in force due to the requirement of the organic certification bodies. Interviewees argue that the combination of restrictions of pesticide use, and cosmetic standards leads to a higher likelihood of FL generation in organic supply chains, as compared to conventional ones.

### 3.4. Limitations

Three main limitations must be considered for this study. First, only three interviews were conducted in the Italian case, and they were not literally transcribed, as it was the case in Germany. However, the Italian POs involved in the study are quite large and relevant at the national level, and full notes were taken during the interviews. Second, interviews can only capture perceptions and knowledge on processes, and therefore enhance the understanding of FL mechanisms. However, the approach is not suitable for assessing quantities of FL. Therefore, we cannot relate the interviewees’ views to the actual quantity of FL the POs generate. Third, and last, it should be remarked that the views reported in the study reflect the perspective of the POs, which may contrast with the perception of the other supply chain actors.

## 4. Conclusions

The aim of this paper is to examine differences and similarities between Italy and Germany considering FL generation in the upstream stages of the supply chain, focusing on the FFV sector. Through a series of qualitative interviews, we gained insights from POs about the organisation of the supply chain, the content of contracts and agreements with buyers, and the relation between FL and private quality standards imposed by buyers. The study confirms the key role of POs in the FFV supply chain. They increase farmers’ bargaining power against retailers and food industry, both in Germany and in Italy. However, retailers still have the power to impose most of the conditions to POs and farmers concerning the terms of contracts and agreements. The content of these agreements shows differences among the two countries analysed, especially concerning the timing with which quantities and prices are defined. Retailers also require private quality standards to POs, which are typically stricter than those imposed by law. This mechanism can generate a significant amount of suboptimal products that are likely to become FL in the absence of alternative marketing channels.

This study suggests that some key antecedents of FL at the upstream stages of FFV supply chains, such as the concentration of the supply, the way private quality standards are enforced by retailers, and the opportunities to reallocate suboptimal products, are quite similar across POs located in Germany and Italy. Nevertheless, several conditions are different, such as levels of informality in Germany, especially in the contract agreements between POs and buyers. This, in turn, may lead to diverging responsibility for the produce, and thus result in varying points within the supply chain where actors can implement food loss prevention measures. An example is the packaging of produce earlier or later in the supply chain, which would require FL reduction measures either in the field or at the PO level, respectively. Overall, this means that POs represent a crucial role in the implementation of FL prevention strategies targeted at the upstream stages of the supply chain. In addition, from the policy perspective, involving the POs in the discussion and implementation of action against FL is crucial, also considering that the same dynamics of FL generation might be found in the FFV sector of other EU countries. The evidence of this study confirms that the whole issue of food loss and waste must be addressed from a broader perspective. Additionally, in the first stages of the supply chain, the framework to address the issue should be set and implemented starting from the EU level and the Common Agricultural Policy instruments.

## Figures and Tables

**Table 1:** Description of the POs involved in the study.

PO	Country	Number of Members	Total Acreage (ha)	Products
PO 1	Germany	19	600	Onions
PO 2	Germany	1200	900	Pomaceous Fruit, Berries, and Asparagus
PO 3	Germany	60	152	Vegetables
PO 4	Germany	330	2500	Pomaceous Fruit
PO 5	Germany	350	5500	Pomaceous Fruit
PO 6	Germany	50	270	Vegetables and Herbs
PO 7	Germany	50	10,000	Vegetables and Herbs
PO 8	Italy	183	1200	Salad, Potatoes, Carrots, and Fennel
PO 9	Italy	85	565	Potatoes, Cabbage, Chickpeas, Beans, and Lentils
PO 10	Italy	4957	32,091	Fruits, Potatoes, and Onions

**Table 2:** Summary of similarities and differences between German and Italian POs, divided by thematic areas.

Thematic Areas	Similarities	Differences
Supply chain organisation	Structure of the FFV supply chain is vertically integrated. Central role of the PO, and as intermediary between farmers and retailers. POs store and prepare the products to be sold. Orders are made with short notice. Flexibility of POs to supply orders.	Selection takes place at different stages of the products (field for Germany; POs’ warehouse in Italy). POs mostly plan the quantity and products to be cultivated by farmers in the next season to better supply orders in Italy; some members have more leeway in product and quantity planning in Germany. Timing and quantities to be delivered are negotiated at the beginning of the season in Italy, informally agreed upon within annual consultations in Germany
Contracts and agreements	POs represent farmers in the commercial relations and negotiations. Buyers take advantage due to their bargaining position. POs usually have contracts with different buyers. Contracts with the food industry are generally considered more reliable (hardly changing quantity and timing that is demanded). Price paid by retailers is higher than the one of the food industries; therefore, retailers are the first choice. Timing and quantity of everyday orders can vary significantly during the season. Final quantities and prices are defined on a short-term basis by retailers and last-minute changes are frequent. Some of the contracts between POs and retailers are on the edge of unfair trading practices (EU legislation). Prices between POs and retailers are negotiated upon weekly or biweekly; influenced by the European and world market prices. Promotions are scheduled by retailers at the beginning of the year, while quantities are announced few weeks before; this is considered a potentially strategic tool to avoid FL.	Economic risks are with different actors of the supply chain. Ownership of the products belongs to POs in Italy, to different stakeholders (POs or farmers) in Germany. Timing, negotiation, and structure of contracts differs between POs and buyers. Differences in the mechanism of the price setting between POs and retailers, and between farmers and POs.
FL and quality standards	Private quality standards are a crucial issue in generating FL. Each retailer asks for different specific quality standards that are generally stricter than the quasi-public and public standards imposed by the EU and UNECE. If private standards are not respected, products are not accepted during the retailer’s quality control and are sent back to the PO, which potentially generates FL. Visual standards, calibres, and pesticide limits are the most difficult private quality standards to be reached. Many alternative marketing channels are available to reallocate suboptimal products. The combination of restrictions to pesticide use and cosmetic standards leads to a higher likelihood of FL generation in organic supply chains compared to conventional ones.	Quality standards are mostly specified in the contracts in Italy; retailers impose these standards in an informal manner (or written form but not contract) in Germany.

## Data Availability

The original data supporting the results of this paper is unavailable due to privacy or ethical restrictions.
